# Role of the tomato *TAGL1* gene in regulating fruit metabolites elucidated using RNA sequence and metabolomics analyses

**DOI:** 10.1371/journal.pone.0199083

**Published:** 2018-06-12

**Authors:** Xiaodan Zhao, Xinyu Yuan, Sha Chen, Lanhuan Meng, Daqi Fu

**Affiliations:** 1 School of Food and Chemical Engineering, Beijing Technology and Business University, Haidian District, Beijing, China; 2 Laboratory of Food Biotechnology, College of Food Science and Nutritional Engineering, China Agricultural University, Haidian District, Beijing, China; 3 Institute of Traditional Chinese Medicine, China Academy of Chinese Medical Sciences, Beijing, China; Universite Paris-Sud, FRANCE

## Abstract

Fruit ripening is a complex biological process affecting fruit quality. In tomato the fruit ripening process is delicately regulated by transcription factors (TFs). Among these, the *TOMATO AGAMOUS-LIKE 1* (*TAGL1*) gene plays an important role in both the development and ripening of fruit. In this study, the *TAGL1* gene was successfully silenced by virus-induced gene silencing technology (VIGS), and the global gene expression and metabolites profiles of *TAGL1*-silenced fruits were analyzed by RNA-sequence analysis (RNA-seq) and liquid chromatography–mass spectrometry (LC-MS/MS). The *TAGL1*-silenced fruits phenotypically displayed an orange pericarp, which was in accordance with the results expected from the down-regulation of genes associated with carotenoid synthesis. Levels of several amino acids and organic acids were lower in the *TAGL1*-silenced fruits than in the wild-type fruits, whereas, α-tomatine content was greatly increased (more than 10-fold) in the *TAGL1*-silenced fruits compared to wild-type fruits. The findings of this study showed that *TAGL1* not only regulates the ripening of tomato fruits, but also affects the synthesis and levels of nutrients in the fruit.

## Introduction

Fruit ripening is a complex developmental process that involves the transformation of the seed-bearing structure of fleshy fruit species into a delicious and nutritive fruit, which appeals to animals and humans, who consume the fruit and act as the dispersers of its seeds [1]. Some general ripening-associated changes are characteristic among different species, including modifications in texture, changes in the sugar content, and alterations in the composition and levels of secondary metabolites such as pigments and flavor [2, 3]. These changes are associated with alterations in multiple biochemical pathways that are regulated by some critical TFs [4, 5].

Tomato (*Solanum lycopersicum*), one of the world’s most important horticultural crops and an important source of human nutrients, is recognized as an outstanding experimental model to study fleshy fruit development and ripening [3, 4]. The tomato fruit is a typical climacteric fruit with a peak of respiration and ethylene production at the start of ripening [6]. Ethylene is synthesized from S-adenosylmethionine (SAM) by the sequential action of two key ethylene biosynthetic enzymes, namely, 1-aminocyclopropane-1-carboxylic acid (ACC) synthase (ACS) and ACC oxidase (ACO) [6,7]. Many transcription factors modulate ethylene synthesis and signal transduction during fruit development and ripening, such as those encoded by the genes *RIPENING INHIBITOR* (*MADS-RIN*) [8], *COLORLESS NON-RIPENING* (*CNR*) [9], and *NON-RIPENING* (*NOR*) [10], and mutations in these genes result in the un-ripened phenotype of the fruit][11]. The RIN protein has been shown to interact with the CArG-box elements in the promoters of genes encoding ACC synthase 2 (ACS2), ACC synthase 4 (ACS4), and cell wall hydrolases such as polygalacturonase (PG), β-galactosidase 4 (TBG4), endo-(1,4)- β- mannanase 4 (MAN4), and α-expansin 1 (EXP1), indicating that it controls ethylene production and fruit softening by directly regulating the transcription of related genes [12,13]. CNR was implicated in the positive regulation of many ripening-related genes, including *polygalacturonase gene (PG)*, *pectinesterase gene (PE)*, *xyloglucan endotransglycosylase gene (XET)*, *phytoene Synthase 1* (*PSY1*), *lipoxygenase* (*LOX*), and *ACC oxidase 1* (*ACO1)* [14]. *NOR* was reported to be involved in the regulation of fruit ripening and quality, possibly by altering the expression of *ACO*, *PSY1*, *PG2*, and others [10]. Other known ripening-associated transcription factors include *TOMATO AGAMOUS-LIKE1* (*TAGL1*) [15, 16, 17, 18], *FRUITFULL* (*FUL1* and *FUL2*) [19, 20], and *APETALA2a* (*AP2a*) [21, 22]. The connections between this highly linked regulatory network and the downstream effectors regulating the color, texture, and flavor of the fruits still remain relatively poorly understood.

*TAGL1* belongs to the *AGAMOUS* clade of the *MADS-box* genes in tomato. Previous studies have revealed that *TAGL1-*RNAi plants produced tomato fruits that were unable to ripen normally, had thin pericarp, and displayed a yellowish-orange color and greater firmness, features that are associated with reduced carotenoids and ethylene levels [15, 16]; over-expressing *TAGL1* resulted swollen sepals and showed ectopic lycopene production and accumulation of the yellow flavonoid naringenin chalcone [16]. The TAGL1 protein is able to bind to the promoter region of *ACS2*, directly regulating the activity of ethylene biosynthesis [16]. Moreover, the pericarp cells of the *TAGL1-*RNAi fruits showed altered cellular and structural properties correlated with the decreased expression of genes regulating both cell division and lignin biosynthesis [23]. However, no study has assessed how *TAGL1* transcription regulates fruit nutrition and flavor, which is the sum of the interactions between sugars, acids, and multiple volatile chemicals. Therefore, the present study was designed with the aim of providing an analysis of *TAGL1* regulation during fruit ripening using RNA sequencing (RNA-seq) and LC-MS/MS.

## Materials and methods

### Plant material and growth conditions

Seeds of the tomato cultivar ‘*Ailsa Craig*’ (AC) were germinated in commercial tomato-cultivation soil. All tomato plants were grown in a greenhouse at 25°C with 75% relative humidity under 16 h light/8 h dark cycles. Flowers were tagged one day post-anthesis (DPA).

### Preparation of vectors

**Fig 1:**
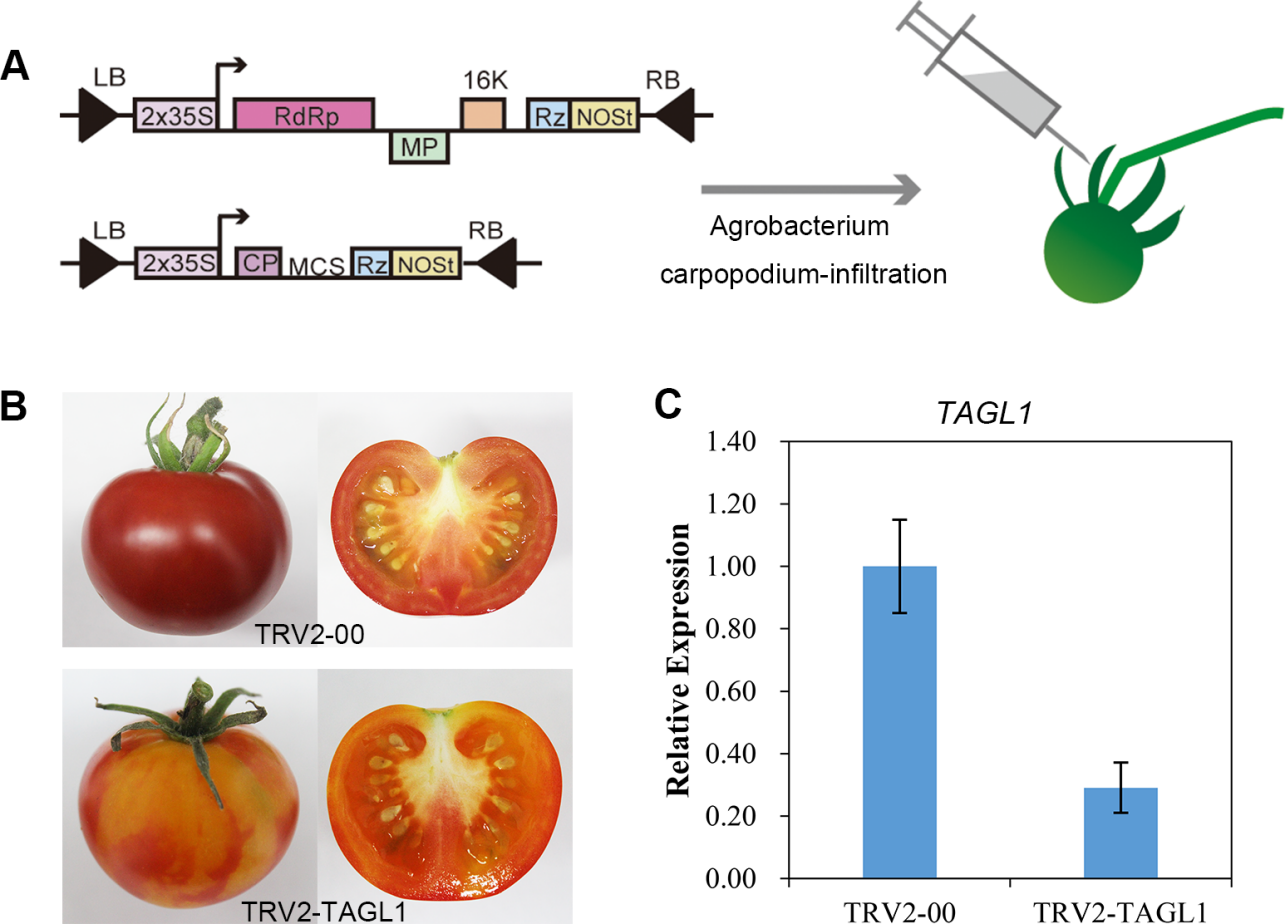
VIGS technique applied to the tomato fruits. (A) Diagram of the VIGS technique used for infecting the tomato fruits. (B) Phenotype of the *TAGL1*-silenced fruits. Fruit infiltrated with the vector (TRV2-00) was used as control. (C) The silencing efficiency of the *TAGL1* gene at the red-ripe stage (RR) using quantitative real time PCR (qRT-PCR).

The tobacco rattle virus (TRV)-based vectors pTRV1 and pTRV2 were adopted for virus-induced gene silencing (VIGS). To construct a pTRV2-*TAGL1* recombinant, a 420 bp *EcoR* I/*BamH* I digested DNA fragment of the *TAGL1* gene corresponding to bases 481–900 of the *TAGL1* gene sequence (NM_001313930.1) were PCR-amplified from tomato cDNA and inserted into the pTRV2 vector (Fig 1A). The VIGS primers are listed in S1 Table.

### Virus-induced gene silencing (VIGS) assay

The VIGS assay was carried out as per the previously described protocol of Fu et al [24] with slight modifications. The *Agrobacterium GV3101* strain containing pTRV1, pTRV2, or pTRV2-*TAGL1* vectors were grown at 28°C in Luria-Bertani medium containing 10 mM MES and 20 mM acetosyringone, with kanamycin, gentamycin, and rifampicin antibiotics. After incubation with shaking for 24 h, the *Agrobacterium* cells were harvested and resuspended in infiltration buffer (10 mM MgCl_2_, 10 mM MES, pH 5.6, 200 mM acetosyringone) and adjusted to a final OD_600_ of 6.0. Resuspensions of pTRV1 and pTRV2 or pTRV2-*TAGL1* were kept for 3–4 h at 25°C and mixed in a ratio of 1:1 before infiltration. Each mixture of the *Agrobacterium* strain was injected into the carpopodium of the tomato fruit on about 7–10 DPA with a 1-ml syringe (Fig 1A). Tomato fruits injected with pTRV1 and pTRV2 alone were used as controls. All fruit samples were harvested at approximately 51 DPA when the *TAGL1* VIGS fruits were at the red-ripe (RR) stage and produced an obvious visible phenotype. Upon harvesting, the yellow pericarps of *TAGL1* silenced fruits were collected, snap-frozen in liquid nitrogen, and stored at -80°C until use.

### RNA-seq and data processing

Total RNA was extracted from the TRV2 control red tomato fruit and the orange pericarp section of *TAGL1-*silenced fruit, (three biological replicates) using an RNeasy MiniKit (Qiagen, GmbH, Germany) at breaker+3 stage. RNA quality was checked on 1% agarose gels. RNA purity and concentration was monitored using a Nano Photometer^®^ spectrophotometer (Implen, CA, USA). Sequencing libraries were generated using NEBNext^®^ Ultra^TM^ RNA Library Prep Kit for Illumina^®^ (NEB, USA) following the manufacturer’s instructions, and index codes were added to attribute each sequence to the respective sample. Library quality was assessed on the Agilent Bioanalyzer 2100 system. According to the manufacturer’s instructions, the clustering of the index-coded samples was performed on a cBot Cluster Generation System using Hiseq 4000 PE Cluster Kit (Illumia, San Diego, CA, USA). After cluster generation, the library preparations were sequenced on an Illumina Hiseq 4000 platform and 150 bp paired-end reads were generated.

RNA data processing was carried out as described previously [25]. In short, raw reads were checked for quality and trimmed to remove barcode and adaptor sequences using Cutadapt (https://pypi.python.org/pypi/cutadapt/) and the FASTX-Toolkit (http://hannonlab.cshl.edu/fastx_toolkit/download.html). The quality of the clean reads was checked using the Q < 20 threshold. All clean reads were deposited in the National Center for Biotechnology Information’s (NCBI) Sequence Read Archive (http://www.ncbi.nlm.nih.gov/sra/) under the accession number SRP076745. Clean reads from each library were aligned to the tomato reference genome (SGN release version SL2.50, ftp://ftp.sgn.cornell.edu/tomato_genome) using TopHat (http://ccb.jhu.edu/software/tophat/index.shtml). To construct the transcripts, reads with less than two mismatches were used by Cufflinks (http://cole-trapnell-lab.github.io/cufflinks/) with the gene annotation of tomato (ITAG2.5). Genes in TAGL1-RNAi and control lines were considered as differentially expressed genes (DEGs) if the fold-change was ≥ 2 and the Q-value was < 0.05 using DESeq2. The averages of gene expressions in the three replicates were used for DEGs identification. Data shown in S3 Table

### Gene Ontology (GO) enrichment analysis and KEGG pathway analysis

WEGO (http://wego.genomics.org.cn/) was used for Gene Ontology (GO) enrichment. The GO enrichment analysis provided all the GO terms that were significantly enriched in the DEGs relative to the genomic background, and the DEGs were filtered according to cellular components, molecular functions, and biological processes. KEGG (Kyoto Encyclopedia of Genes and Genomes; http://www.genome.jp/kegg/) is a main pathway-related database. Based on the comparison of the DEGs to the genomic background, pathway enrichment analysis pinpointed the enriched pathways.

### Validation of RNA-Seq by qRT-PCR

2 μg total RNA was reverse-transcribed into cDNA with cDNA Synthesis SuperMix (TransGen Biotech, Beijing, China) with oligo (dT) primers, and the genomic DNA was removed using TranScript one-step gDNA Removal. Quantitative real-time PCR (qRT-PCR was performed using SYBR Green PCR SuperMix (Trans, China) with a BIO-RAD real-time PCR System CFX96 (Bio-Rad, U.S.A). The reaction condition was set as follows: 95°C for 10 min, followed by 40 cycles of 95°C for 15 s and 60°C for 30 s. The fluorescence signal was monitored automatically in each cycle. Relative expression levels of specific mRNAs were measured using the 2^(-ΔΔCt) analysis method, and the expression values were normalized using the *Actin* gene. For each sample, three independent biological replicates were analyzed. Primers used in this validation are listed in the S1 Table.

### Metabolite extraction for LC-MS analysis

Metabolite extraction was performed following the method described previously by Chen et al. with some modification [26]. The frozen pericarp samples were ground into powder using a mortar and pestle. 100 mg powder was weighed and suspended in 1.0 mL pure methanol or 75% aqueous methanol for extraction of lipid-soluble metabolites or water-soluble metabolites, respectively, both containing 20 mg/L lidocaine and 20 mg/mL CHAPS. Suspensions were vortexed 1 min for three times for and then stored at 4°C overnight. Following centrifugation at 12000 rpm for 10 min, supernatants containing the lipid-soluble metabolites and water-soluble metabolites were collected and mixed in a ratio of 1:1, and then filtered before LC-MS analysis.

### Instrumentation and chromatographic system

A high-performance liquid chromatography (HPLC-20A) unit equipped with a photodiode array detector (Shimadzu, Japan) was used to analyze the metabolites in the tomato extracts. Separation of the metabolites was performed under the following conditions: column, Eclipse XDB-C_18_ (3.0*50 mm); solvent system water (0.2% formic acid); acetonitrile; gradient program, 95:5V/V at 0 min, 5:95V/V at 12 min, 5:95V/V at 15 min, 95:5 at 15.1 min, 95:5V/V at 22 min; flow rate, 0.2 ml/min; temperature: 45°C; injection volume: 2 μL. Masses of the eluted compounds that ranged from 50 m/z to 1500 m/z were monitored with an Agilent 6460 triple quad LC-MS equipped with an ESI source.

Quantitative detection was performed using UHPLC-ESI-QQQ-MS (Agilent 1290 and 6460 Triple Quadrupole Mass Spectrometry Series, Agilent Corporation, CA, USA). An electrospray ionization (ESI) source working either in positive or negative ion mode was used for all mass spectrometry (MS) analyses, using nitrogen as the drying agent. The MS conditions in the positive mode were as follows: HV voltage 4000 kV; capillary 7 μA, nozzle voltage 500 V, delta EMV 300V, gas flow 5 L/min; gas temperature 400°C; sheath gas flow 11 L/min. Collision energy was optimized based on the standards. Helium was used as the collision gas for collision-induced dissociation (CID). Quantification was done using the multiple reaction monitoring (MRM) mode under unit mass-resolution conditions. The data recorded were processed with MassHunter Software.

### Chemicals

MS-grade acetonitrile and methanol were obtained from Sigma-Aldrich (St. Louis, MO, USA). Formic acid (eluent additive used for HPLC-MS analysis) was MS grade (CNW, Germany). The remaining analytical-grade chemicals were obtained from Beijing Chemical Factory (Beijing, China).

## Results and discussion

### Silencing of *TAGL1* gene inhibited fruit ripening

In order to obtain *TAGL1*-silenced tomato fruit by VIGS, *Agrobacterium* GV3101 cultures containing either pTRV1: pTRV2-*TAGL1* or pTRV1 and pTRV2-00 were injected into the carpopodium of the tomato fruit 7–10 DPA after pollination and fruit phenotypes were observed 20–30 days after injection. When all control fruits injected with pTRV1 and pTRV2-00 had turned red, thirty fruits at the same stage that had been injected with pTRV1 and pTRV2-*TAGL1* showed an obviously different phenotype, with orange and red colored regions on the same fruit (Fig 1B). To confirm that the *TAGL1* gene was suppressed successfully at the molecular level in *TAGL1*-silenced fruit, primers specific to the *TAGL1* gene outside the region targeted for silencing were designed for real-time PCR. Reverse transcription -PCR showed that the *TAGL1* transcripts in the orange sections of the *TAGL1*-silenced fruits were reduced by more than 71% compared to the red control fruits infiltrated with empty TVR vector (Fig 1C). The result reveals that the down-regulation of *TAGL1* causes the unusual ripening phenotype of tomato fruits, which was consistent with the previous finding that *TAGL1*-RNAi fruits were yellow-orange upon ripening [15, 16]. However, in our experiments, *TAGL1*-silenced fruits did not show a thin pericarp layer phenotype displayed by RNAi-*TAGL1* transgenic fruits [16]. A previous study has reported that the highest expression of the *TAGL1* transcripts was observed during flower anthesis and the red ripe stage of tomato fruit [15, 16, 17]. As we injected tomato fruit 7–10 days after pollination, there was no opportunity for *TAGL1* expression to be silenced in the tomato flower. We speculate, therefore, that *TAGL1* may regulate pericarp development at the stage of ovary development rather than fruit ripening.

### Global overview of the RNA-seq profile of *TAGL1*-silenced tomato

**Fig 2:**
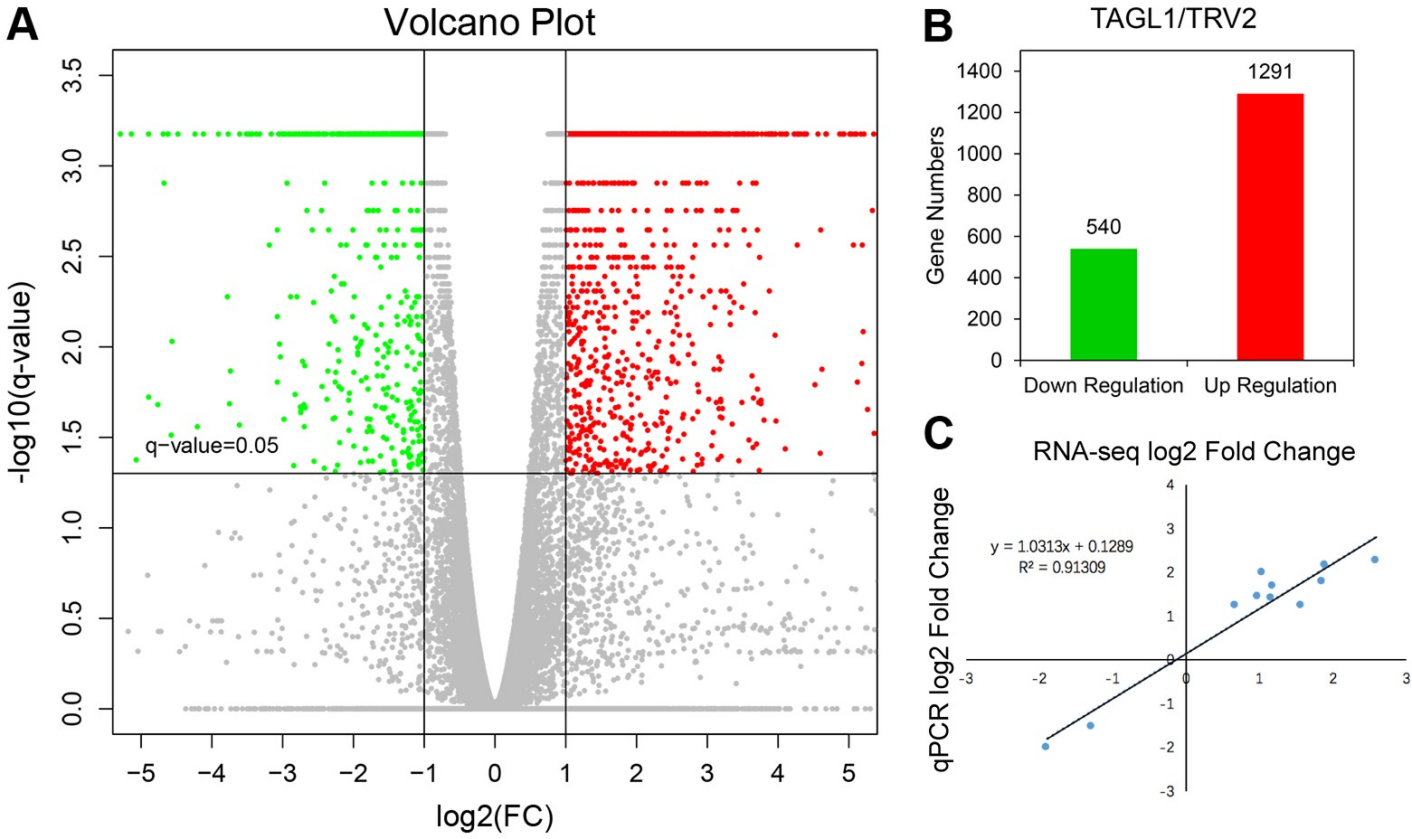
Global view of the DEGs in the *TAGL1*-silenced tomato fruits. Volcano diagrams of the DEGs. Spots above the threshold line (*Q*-value = 0.05), indicate that differences are significant. Genes with expressions less than half of that displayed in the control group for *Q*-value < 0.05 are displayed in the green area, while those with expression at least two-folds greater than that of the control group are displayed in the red area. Genes in the grey area were neither over- nor under-expressed. (B) The number of down/up regulated genes. (C) The expression levels of 11 genes, as determined by RNA-seq and qPCR, are closely correlated. Nine genes were up-regulated, while two genes were down-regulated.

To understand the role of *TAGL1* in regulating tomato fruit ripening at the molecular level, *TAGL1*-silenced (orange section) and control fruit samples(red stage) (TRV2-00) were analyzed via RNA-seq. All clean reads generated in the sequencing experiments were mapped and aligned with the tomato reference genome (ITAG2.5). Within each file, 78.38 ± 1.92% of the reads were found to be uniquely aligned, suggesting that the sequencing results were relatively stable (data not shown). Using the cutoff criteria of expression ratio ≥2.0 and Q-value < 0.05 (BH correction) between *TAGL1*-silenced and control tissues, 1291 up-regulated and 540 down-regulated genes were identified in *TAGL1*-silenced samples compared to controls (S3 Table and Fig 2). These results indicate that silencing *TAGL1* affects the expression of many genes. To validate RNA-Seq results, 11 ripening or nutrition-associated DEGs were selected and their expression levels were verified by qRT-PCR. The fold changes in the expression patterns of the DEGs in real-time PCR were very similar (r^2^ = 0.91) (Fig 2, S1 Fig).

**Fig 3:**
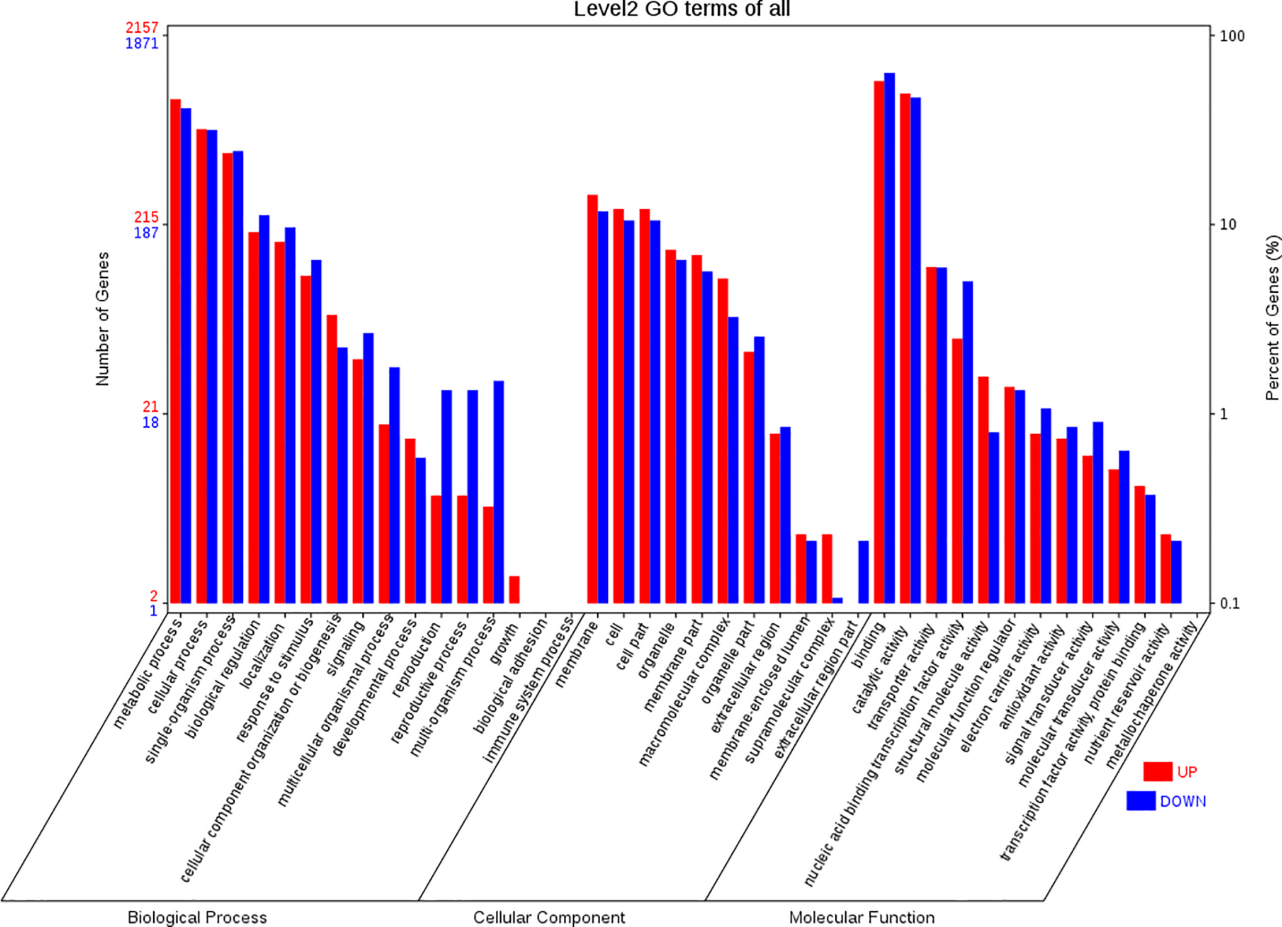
GO analysis classified DEGs in the *TAGL1*-silenced tomato fruits according to WEGO.

**Fig 4:**
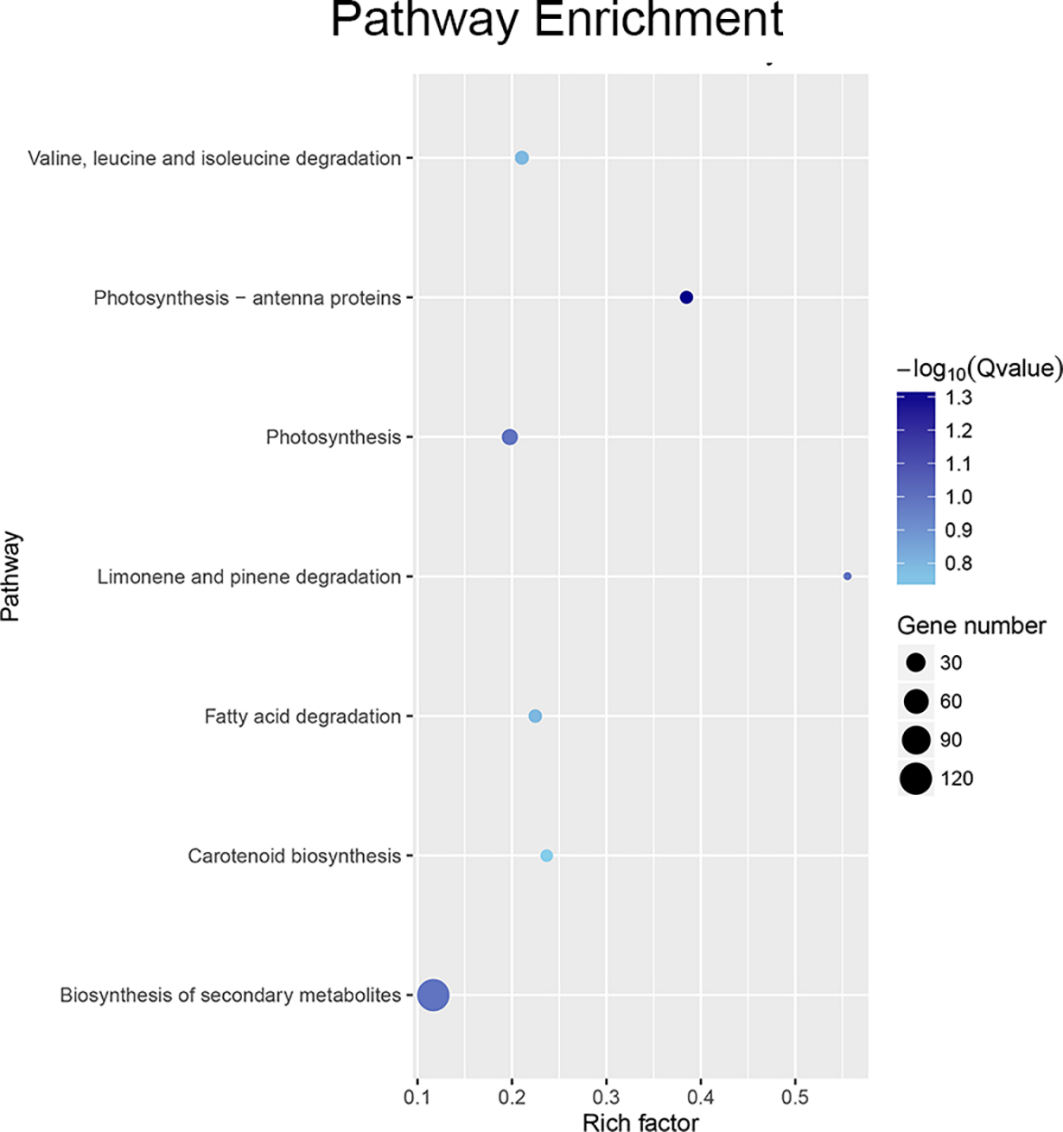
Pathway enrichment analysis of differentially displayed genes from the *TAGL1*-silenced tomato fruits.

Gene ontology was successfully assigned using WEGO. The SDEGs between the TRV2-*TAGL1* and the TRV2-00 were classified into 40 functional groups (Fig 3), of which cellular components accounted for 11 GO terms (the most representative were “membrane” and “cell”), molecular functions accounted for 13 GO terms (the most representative was “binding” and “catalytic”), and biological processes accounted for 16 GO terms (the most representative were “metabolic process”). Results of the KEGG pathway enrichment analysis indicated that *TAGL1* plays a role in photosynthesis and biosynthesis of secondary metabolites (Fig 4).

### Analysis of constituents in tomato fruit samples

In order to observe the effects of *TAGL1* gene silencing on the tomato fruit at the metabolic levels, the presence of 50 chemical compounds were tested and quantified in positive and/or negative modes. The identified compounds were classified into four groups: amino acids, organic acids, phenolics, and solanum alkaloids. By comparing the UPLC retention times and mass spectral data with those of the reference standards, the target peaks were tentatively identified as described below. The metabolites for which commercial standards are unavailable were identified following previously described protocols [26]. Peaks were used to query the MS^2^ spectral data taken from the literature or to search the databases (MassBank, Moto DB, KOBAS). Best matches were then searched in the Dictionary of Natural Products (DNP) and KEGG for identifying possible structures. More than 20 metabolites were putatively identified.

### Silencing of *TAGL1* altered fruit metabolism

In order to evaluate the changes in the nutrition and flavor of the *TAGL1-*silenced fruits, metabolites were analyzed using LC-MS/MS. Fifty metabolites were detected and annotated using standards and/or cross-referencing against libraries, and the production level of 11 metabolites were found to be different between TRV2-*TAGL1* (orange section) and TRV2-00 samples (red) (Table 1).

**Table 1:** Relative quantitation of metabolites in *TAGL1*-silenced tomato fruits.

Analytes	Ratio (TAGL1/TRV2)	P-value	Pathway
Aspartic acid^1^	0.467631696	0.014933727	Biosynthesis of amino acids
L-Tyrosine^1^	0.202144376	0.015775349	Biosynthesis of amino acids
Feruloylputrescine	0.153963685	0.014338758	Arginine and proline metabolism
5-caffeoylquinic acid	0.468658398	0.001019251	-
α-Tomatine^1^	10.78557867	8.33493E-08	-
L-Phenylalanine^1^	0.14565209	0.027272425	Biosynthesis of amino acids
L-Valine^1^	0.449745752	0.011035025	Valine, leucine, and isoleucine degradation
L-Glutamic acid^1^	0.244228151	3.67814E-06	Biosynthesis of amino acids
Isoleucine	0.26956715	0.015007356	Valine, leucine, and isoleucine degradation
L-Leucine^1^	0.203396442	0.012507063	Valine, leucine, and isoleucine degradation
C11H23O12P	0.258873555	0.000509657	-
C34H46O14	1.5902	1.5073E-06	-
4-Aminobutanoic acid^1^	0.456910298	0.001414631	Arginine and proline metabolism

^1^ The analytes were identified by comparison with standards.

Amino acid content of tomato fruits contributes markedly to the taste and nutritional quality. The data showed that the level of seven amino acids dropped in the *TAGL1*-silenced tomato fruits, with aspartic acid, L-tyrosine, L-glutamic acid, L-phenylalanine, Lvaline, L-leucine^1^, and isoleucine in the TRV2-*TAGL1* fruits reduced to 46.76%, 20.21%, 24.42%, 14.57%, 44.97%, 20.34%, and 26.96% of that in the control fruits, respectively (Table 1). Aspartic acid and glutamic acid are known as amino acids that confer a delicious flavor; phenylalanine and tyrosine are known aromatic amino acids; and leucine, isoleucine, and phenylalanine are essential amino acids for humans. The results indicate that the down-regulation of the *TAGL1* gene leads to the reduction in the content amino acid in the tomato fruit.

**Fig 5:**
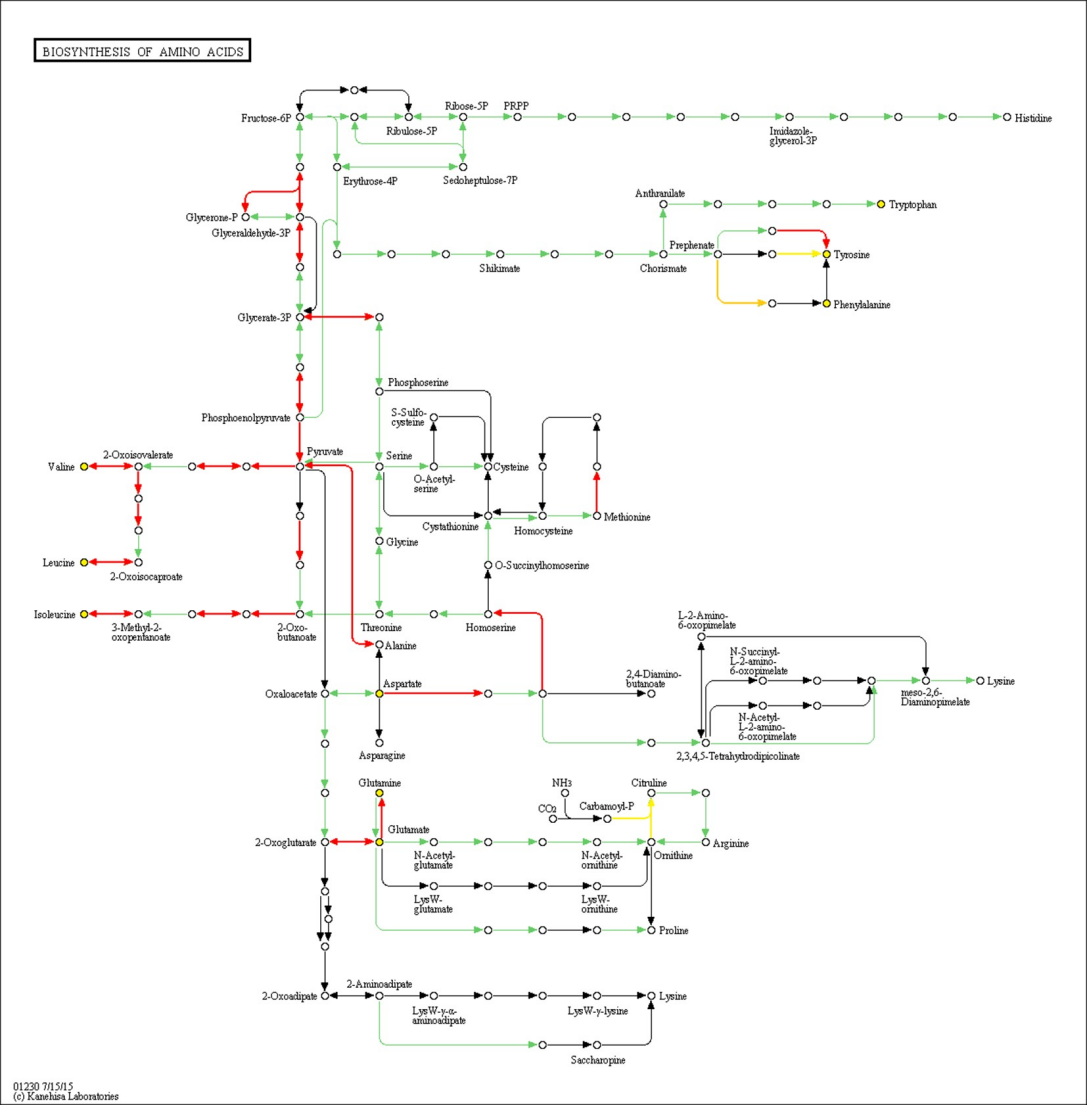
Diagram of amino acid biosynthetic pathways.

To understand the mechanism of *TAGL1*’s regulation of amino acid synthesis, gene expression profiles were analyzed, and 20 DEGs were found to be enriched in the pathway of biosynthesis of the amino acids (Fig 5 and S2 Table). Only three of these genes have been reported before, including *gs* (encoding glutamine synthetase (GS)) [27], *SAM3* (encoding S-adenosylmethionine (SAM) synthase 3) [28], and *IPMS2* (encoding 2-isopropylmalate synthase) [29]. GS is a type of chloroplast glutamine synthetase that assimilates ammonia into glutamine, which is a metabolic intermediate in the synthesis of other nitrogen-containing compounds in the plant. In addition, SAM3 catalyzes the formation of S-adenosylmethionine from methionine and could be induced by ozone exposure [30]. *IPMS2* is one of a cluster of three genes known to encode an enzyme involved in leucine biosynthesis [31]. Among the other unreported genes, only three were down-regulated, including genes that encode prephenate dehydratase (Solyc11g066890) (probably involved in the first step of the sub-pathway that synthesizes L-phenylalanine from L-arogenate), tyrosine aminotransferase-like protein (Solyc07g053720), and ornithine carbamoyltransferase (Solyc12g089210). While L-glutamic acid was reduced to 24.42% of the control fruits in the *TAGL1*-silenced fruits, methionine and ornithine were not detected in either the *TAGL1*-silenced fruits or the control fruits, using LC-MS/MS. Glutamine synthetase catalyzes glutamic acid synthesis from glutamine. From RNA-seq, we found that the expression level of glutamine synthetase gene (Solyc01g080280) was increased about 6-fold in *TAGL1*-silenced fruit compared with control (S2 Table) which reduced the accumulation of L-Glutamic acid in *TAGL1*-silenced tomato fruit (Table 1). It is also possible that translational regulation could possibly explain the lack of positive correlation between gene expressions and level of amino acids. Fourteen DEGs were enriched in the pathway of phenylalanine metabolism using KEGG (S2 Table). These results show that *TAGL1* positively regulates the biosynthesis of several amino acids through a complex network.

The diagram was constructed using KEGG pathway enrichment. Spots represent metabolites and arrows indicate steps. The arrows colored red, yellow, and grey/green indicate up-regulation, down-regulation, and absence of significant differences, respectively.

γ-Aminobutyric acid (GABA), identified as a functional component in reducing blood pressure in the human body, is a four-carbon non-protein amino acid commonly found in bacteria, animals, and plants [32, 33]. GABA is metabolized through a short pathway, called the ‘GABA shunt,’ which is a bypass of the tricarboxylic acid (TCA) cycle, composed of three enzymes, glutamate decarboxylase (GAD), GABA transaminase (GABA-T), and succinic semialdehyde dehydrogenase (SSADH) [34]. The first enzyme, GAD, catalyzes the irreversible decarboxylation of glutamate to produce GABA and CO_2_; then GABA is reversibly transaminated by the second enzyme, GABA-T, to form succinic semialdehyde (SSA), which is oxidized by the third enzyme, SSADH, to produce succinate [35]. The resulting succinate then flows into the TCA cycle. In tomato, GAD2 and GAD3 catalyze GABA synthesis from glutamate; silencing this gene using transgene technology can effectively reduce GABA levels compared with the control [36]. In our study, GABA was reduced to 46% and L-glutamine was reduced to 24% in the *TAGL1*-silenced fruits compared to the controls. In addition, the expression level of glutamate decarboxylase (*GAD3*) (Solyc05g052100) was decreased in the *TAGL1*-silenced fruits, as evidenced by the RNA-seq data. Although the decreased levels of glutamate contribute to the reduction of GABA, the findings of this study indicate that the expression level of *GAD3* is regulated by *TAGL1* to control GABA biosynthesis.

5-caffeoylquinic acid (chlorogenic acid) is one of the most abundant and widespread soluble phenolics in vascular plants. Besides indications that it can protect plant cells against oxidative stress, it can also play a role in the resistance to phytopathogens. 5-caffeoylquinic acid biosynthesis in fruits of the family *Solanaceae* (tomato, tobacco, and potato) was initially thought to occur via transesterification from caffeoyl-CoA and quinic acid by the hydroxycinnamoyl-CoA: quinate hydroxycinnamoyl transferase hydroxycinnamoyl quinic acid [37, 38]. The existence of another route involving direct 3′-hydroxylation of p-coumaryol quinic acid was first suggested by work with carrot cell cultures, and studies of the impact of the level of expression of the hydroxycinnamoyl quinic acid gene in tobacco and tomato plants demonstrated that this route might be predominant in the plants of the *Solanaceae* family [39].

Analysis of the tomato fruits with VIGS-silenced *TAGL1*, revealed a significant reduction in 5-caffeoylquinic acid to 47% of the control fruit. Our RNA-seq data showed that the transcript levels of one gene involved in the first proposed pathway for the synthesis of chlorogenic acid in plants, phenylalanine ammonia lyase gene (*PAL*) (Solyc10g086180), was increased. *PAL* encodes a protein that is involved in the first step of the sub-pathway that synthesizes trans-cinnamate from L-phenylalanine. We conclude that the decreased level of 5-caffeoylquinic acid content is mainly due to a significant reduction in the amount of phenylalanine, its precursor.

α-tomatine found in tomato is a type of antinutritional factor for humans. However, α-tomatine may also have anticarcinogenic, cardioprotective, and other beneficial effects [39]. There exists a proposed pathway for conversion from cholesterol to α-tomatine: GAME7 (CYP72) hydroxylates the cholesterol at the C22 position, followed by GAME8 (CYP72) hydroxylation at C26 position, and then C22, 26-dihydroxycholesterol is hydroxylated at C16 and oxidized at C22, follow by closure of E-ring by GAME11 (2-oxoglutarate-dependent dioxygenase) and GAME6 (CYP72) to form the furostanol-type aglycone [40]. In our study, the level of α-tomatine was significantly increased in the *TAGL1*-silenced fruits (10.49 fold). At the same time, the expression level of *GAME11* (Solyc02g062490) was increased in the *TAGL1*-silenced fruit compared to control tomato fruit. It has been reported, using the VIGS technology to silence *GAME11*, that a putative dioxygenase in the cluster results in a significant reduction in α-tomatine levels and accumulation of several cholestanol-type steroidal saponins in tomato leaves [40], which was consistent with the findings of our study. In summary, *TAGL1* VIGS promotes α-tomatine biosynthesis by activating the expression of *GAME11*.

## Conclusion

Gene silencing of *TAGL1* in tomatoes using the VIGS technique resulted in a non-ripening phenotype with orange pericarp. The analysis of the metabolites by LC-MS/MS showed the reduction in content of several amino acids and organic acids, as well as the accumulation of *α*-tomatine in the *TAGL1*-silenced fruits. The result show that *TAGL1* positively regulates the synthesis of amino acids and negatively regulates the synthesis of α-tomatine in tomato fruit. The findings of the present study suggest that *TAGL1* controls accumulation of nutritional and flavor components in the tomato fruits by the transcriptional regulation of targeted genes.

## Supporting information

S1 Fig Real-time PCR of some candidate genes in tomato fruit. (TIF)

S2 Fig Schematic diagram of phenylalanine biosynthetic pathway. (TIF)

S1 Table Oligonucleotide primers used in the study. (DOCX)

S2 Table Representative DEGs in *TAGL1*-silenced tomato fruits. (DOCX)

S3 Table RNA-seq of *TAGL1 RNAi* and WT at break+3 stage. (CSV)
