# Supplementary figures and images for: Role of the tomato TAGL1 gene in regulating fruit metabolites elucidated using RNA sequence and metabolomics analyses
Source: PLoS One. 2018 Jun 12;13(6):e0199083. doi: 10.1371/journal.pone.0199083 (PMC5997326; doi:10.1371/journal.pone.0199083)

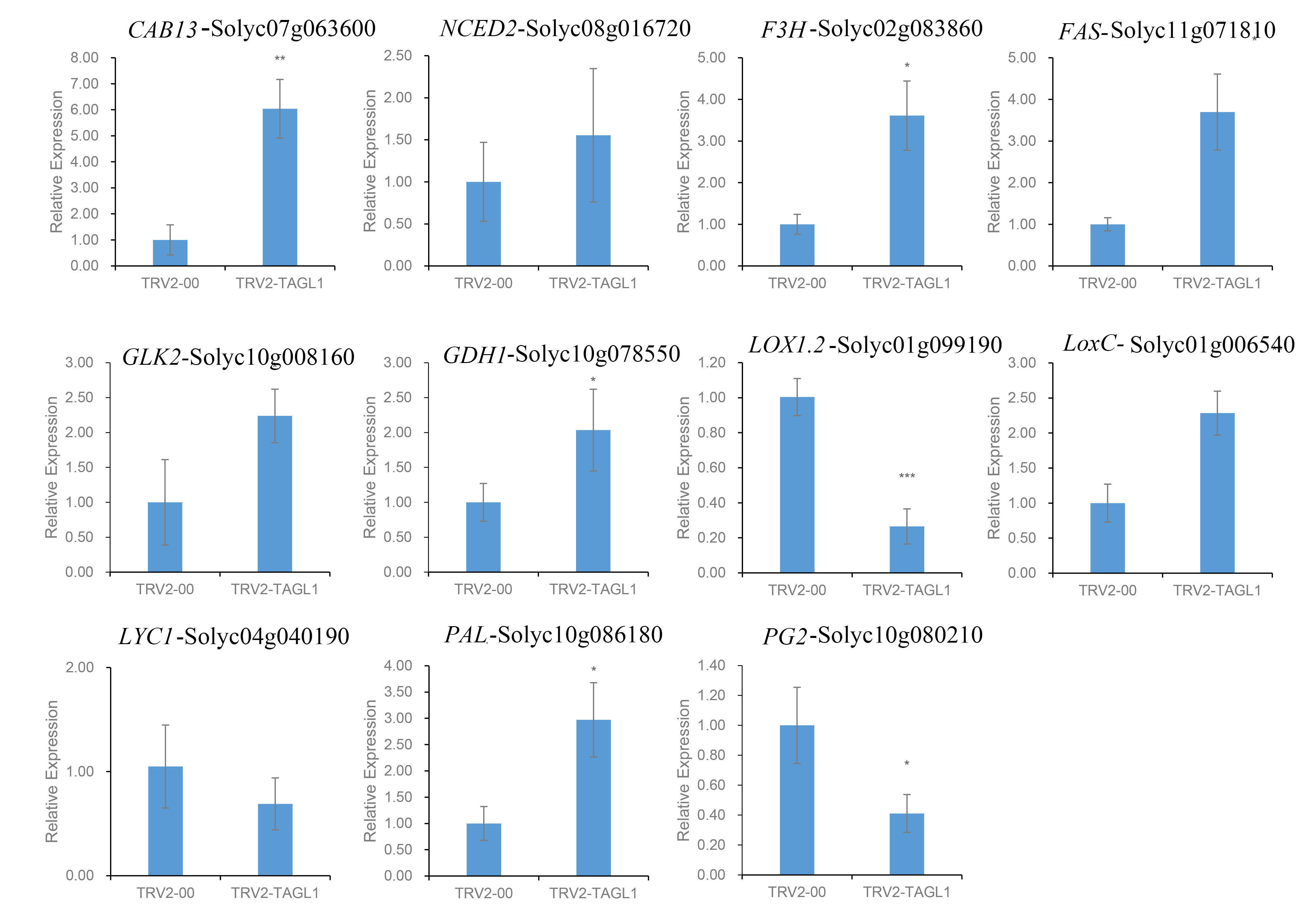

Supplement: S1 Fig — (TIF) [file pone.0199083.s001.tif]

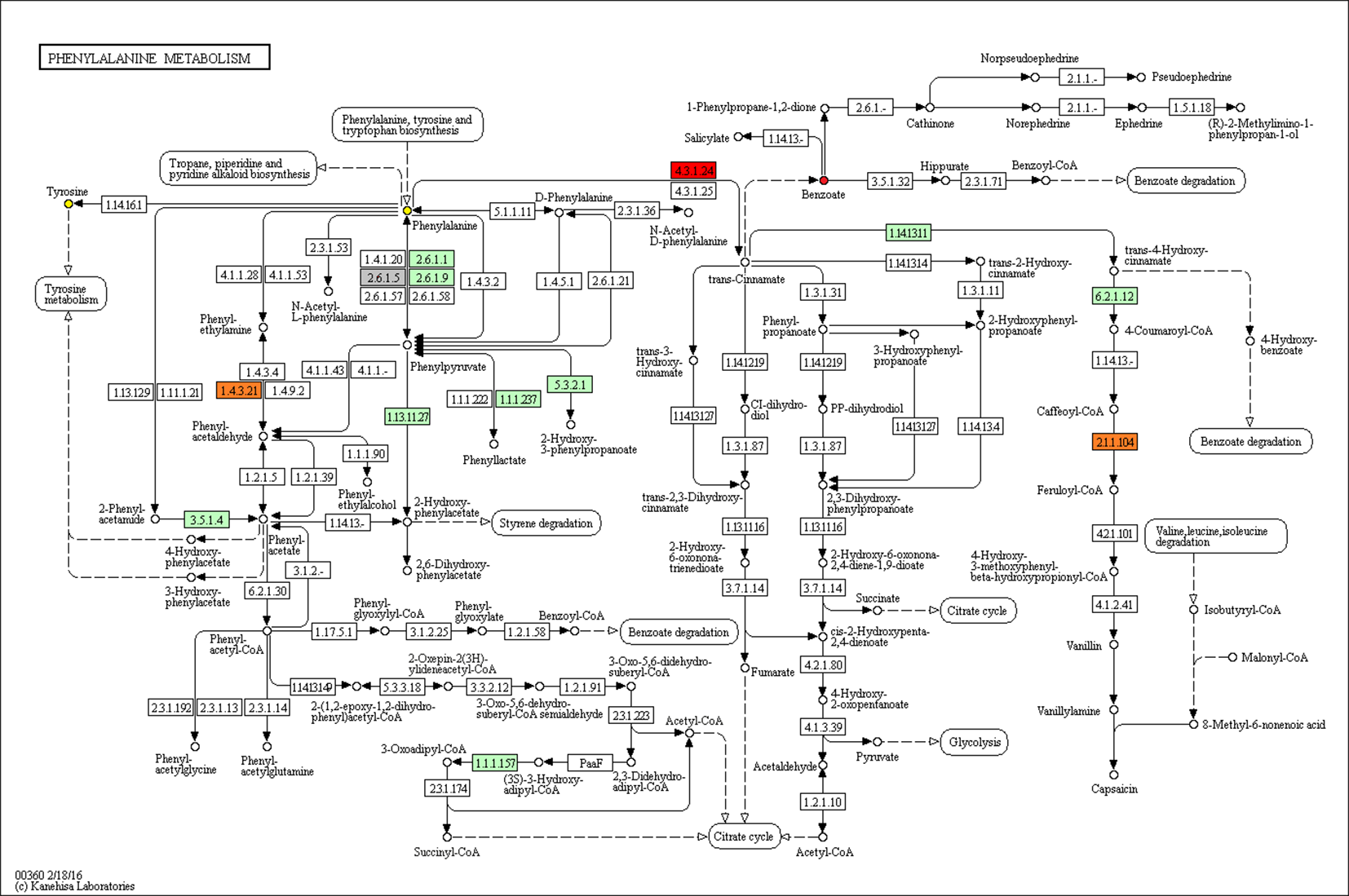

Supplement: S2 Fig — (TIF) [file pone.0199083.s002.tif]
