# Supplementary material for: Role of the tomato TAGL1 gene in regulating fruit metabolites elucidated using RNA sequence and metabolomics analyses
Source: PLoS One. 2018 Jun 12;13(6):e0199083. doi: 10.1371/journal.pone.0199083 (PMC5997326; doi:10.1371/journal.pone.0199083)
Supplement: S1 Table — (DOCX) [file pone.0199083.s003.docx]

| **S1 Table**  **Oligonucleotide primers used in the study.** | |
| --- | --- |
| **Primer** | **Sequence** |
| qPCR-Actin-F | CAGCAGATGTGGATCTCAAA |
| qPCR-Actin-R | CTGTGGACAATGGAAGGAC |
| qPCR-CAB13 (Solyc07g063600)-F | CTCTTGAGGTTATCCATGGGAG |
| qPCR-CAB13 (Solyc07g063600)-R | TTGCCCAAATAGTCCAGCC |
| qPCR-F3H (Solyc02g083860)-F | CCACTTACAGGGTGAAGTGG |
| qPCR-F3H (Solyc02g083860)-R | TCTTTCTCTAAGCCCATTGCC |
| qPCR-FAS (Solyc11g071810)-F | CTTCGATTCGTCCACCAGAG |
| qPCR-FAS (Solyc11g071810)-R | TCCAGCTTGAGTCCAAAGTG |
| qPCR-GDH1 (Solyc10g078550)-F | GAGTGGGTCCAGAACATCCA |
| qPCR-GDH1 (Solyc10g078550)-R | CATCCTCGAAGAACGGTTGC |
| qPCR-GLK2 (Solyc10g008160)-F | TCAAAGTTGACACAGTTGCC |
| qPCR-GLK2 (Solyc10g008160)-R | CACACTGTCAATTGATGGAGGT |
| qPCR-LOX1.2 (Solyc01g099190)-F | TCATTAGCCACTGGTTGAATACAC |
| qPCR-LOX1.2 (Solyc01g099190)-R | GATGAAGCACACTTAGATGCCT |
| qPCR-loxC (Solyc01g006540)-F | AGCTATGGAGGCTACTTTCCA |
| qPCR-loxC (Solyc01g006540)-R | ATTCAAGAACCACTCCCATTCC |
| qPCR-NCED2 (Solyc08g016720)-F | CAAGGTTCGGAATTCTACCCA |
| qPCR-NCED2 (Solyc08g016720)-R | GGTTCTTCCCAAGCATTCCA |
| qPCR-PAL (Solyc10g086180)-F | CTTATCAGGTTCTTGAATGCTGG |
| qPCR-PAL (Solyc10g086180)-R | GCAGAGTATTGATCCTAACAAGC |
| qPCR-PG2 (Solyc10g080210)-F | AGGCTTTGGATTGCTTTTGA |
| qPCR-PG2 (Solyc10g080210)-R | AGAAGGTTAAGGCCGTTGGT |
| qPCR-LYC1  (Solyc04g040190)-F | AGTTCTTCTGCTTCGGTATGG |
| qPCR-LYC1  (Solyc04g040190)-R | GAGACGATAAGAAGCCATGCC |
| qPCR-TAGL1-F | CAGTACTACCAGCAAGAAGC |
| qPCR-TAGL1-R | AGATACATGTTGGCGTTCTG |
| VIGS-TAGL1-F | CTGCTTGGATCCCCCTTAGTCCAAAGTCTCTTCC |
| VIGS-TAGL1-R | GACTTAGAATTCGGAGGCTTCTTGCTGGTAAT |
|  |  |
