# Supplementary material for: Role of the tomato TAGL1 gene in regulating fruit metabolites elucidated using RNA sequence and metabolomics analyses
Source: PLoS One. 2018 Jun 12;13(6):e0199083. doi: 10.1371/journal.pone.0199083 (PMC5997326; doi:10.1371/journal.pone.0199083)
Supplement: S2 Table — (DOCX) [file pone.0199083.s004.docx]

**S2 Table**

**Representative DEGs in *TAGL1*-silenced tomato fruits.**

| **Accession** | **Annotation** | **Ratio** |
| --- | --- | --- |
| **Photosynthesis-antennaproteins** | |  |
| Solyc06g063370 | Chlorophyll a-b binding protein 1A, chloroplastic | 1.62011 |
| Solyc03g005760 | Chlorophyll a-b binding protein 3C-like | 5.01555 |
| Solyc02g071030 | Chlorophyll a/b binding protein | 3.69658 |
| Solyc07g063600 | Chlorophyll a-b binding protein 13, chloroplastic | 2.23882 |
| Solyc07g047850 | Chlorophyll a-b binding protein 4, chloroplastic | 2.22067 |
| Solyc10g006230 | Chlorophyll a-b binding protein 7, chloroplastic | 2.14728 |
| Solyc01g105030 | Chlorophyll a-b binding protein, chloroplastic | 2.63087 |
| Solyc10g007690 | Chlorophyll a-b binding protein 8, chloroplastic | 2.07223 |
| Solyc01g105050 | Chlorophyll a-b binding protein, chloroplastic | 2.64363 |
| **Photosynthesis** |  |  |
| Solyc03g095880 | Unknown Protein | 1.64744 |
| Solyc05g016120 | Photosystem Q | -1.46635 |
| Solyc08g006930 | Photosystem I reaction center subunit X psaK | 3.49274 |
| Solyc09g063130 | Photosystem I reaction center subunit IV A | 2.4328 |
| Solyc08g013670 | Photosystem I reaction center subunit | 5.2039 |
| Solyc06g082950 | Photosystem I reaction center subunit XI | 2.18961 |
| Solyc06g082940 | Photosystem I reaction center subunit XI | 2.70283 |
| Solyc02g079950 | Oxygen-evolving enhancer protein 3 | 2.53657 |
| Solyc06g074200 | Sex-linked protein 9 | 5.61399 |
| Solyc09g065910 | Photosystem II reaction center W protein | 1.55959 |
| Solyc02g065400 | Oxygen-evolving enhancer protein 1 of photosystem II | 1.04452 |
| Solyc02g080540 | ATP synthase gamma chain | 1.69619 |
| Solyc02g090030 | Oxygen-evolving enhancer protein 1 of photosystem II | 2.15789 |
| Solyc12g056830 | ATP synthase delta subunit | 1.40306 |
| Solyc07g066150 | Photosystem I reaction center subunit V | 1.6774 |
| Solyc07g066310 | photosystem II polypeptide | 1.08728 |
| Solyc06g083680 | Photosystem I reaction center subunit IV A | 1.73001 |
| Solyc02g069450 | Photosystem I reaction center subunit III | 1.21174 |
| **Fatty acid degradation** |  |  |
| Solyc04g074530 | Alcohol dehydrogenase 1 | 1.56656 |
| Solyc02g086970 | Aldehyde dehydrogenase 1 | -1.96627 |
| Solyc08g083280 | Alcohol dehydrogenase 2 | 3.04359 |
| Solyc09g092450 | Long-chain-fatty-acid CoA ligase | 1.15452 |
| Solyc01g079240 | Long-chain-fatty-acid--CoA ligase family protein | 1.44488 |
| Solyc03g114150 | Aldehyde dehydrogenase | 1.98487 |
| Solyc10g085200 | Acyl-CoA dehydrogenase | 1.38327 |
| Solyc01g095750 | Long-chain-fatty-acid-CoA ligase | 1.8113 |
| Solyc05g005700 | Aldehyde dehydrogenase 1 | 1.92107 |
| Solyc01g109180 | Long-chain-fatty-acid-CoA ligase | 1.01409 |
| Solyc06g060250 | Aldehyde dehydrogenase family protein expressed | 1.0453 |
| **Valine, leucine and isoleucine degradation** | |  |
| Solyc07g021630 | Branched-chain-amino-acid aminotransferase | 1.71365 |
| Solyc04g063320 | Unknown Protein | 2.84503 |
| Solyc05g056480 | Pyruvate dehydrogenase E1 component alpha subunit | 1.4397 |
| Solyc01g108030 | Methylcrotonoyl-CoA carboxylase beta subunit | 1.41372 |
| Solyc04g063350 | 3-methyl-2-oxobutanoate dehydrogenase | 3.89738 |
| Solyc03g114150 | Aldehyde dehydrogenase | 1.98487 |
| Solyc02g086970 | Aldehyde dehydrogenase 1 | -1.96627 |
| Solyc03g043880 | Branched-chain amino acid aminotransferase | 1.68942 |
| Solyc05g005700 | Aldehyde dehydrogenase 1 | 1.92107 |
| Solyc05g053100 | Dihydrolipoyl dehydrogenase | 2.36309 |
| Solyc06g060250 | Aldehyde dehydrogenase family protein expressed | 1.0453 |
| **Carotenoid biosynthesis** | |  |
| Solyc12g056600 | Short chain alcohol dehydrogenase | 3.0463 |
| Solyc04g078900 | Cytochrome P450 | -2.67018 |
| Solyc04g050930 | Violaxanthin de-epoxidase | 1.26253 |
| Solyc04g040190 | Lycopene beta-cyclase 1 | 1.41635 |
| Solyc11g071600 | Aldehyde oxidase | 3.86192 |
| Solyc01g009230 | Xanthine dehydrogenase/oxidase | 1.07613 |
| Solyc08g016720 | 9-cis-epoxycarotenoid dioxygenase 5 | 1.2343 |
| Solyc08g075320 | Cytochrome P450 | 2.60954 |
| Solyc07g056570 | 9-cis-epoxycarotenoid dioxygenase | 1.23583 |
| **Glutathione metabolism** | |  |
| Solyc07g056500 | Glutathione transferase | -1.17381 |
| Solyc09g063150 | Glutathione S-transferase | 1.72942 |
| Solyc04g026030 | Spermidine synthase | -1.24424 |
| Solyc01g099590 | Glutathione-S-transferase | -1.2961 |
| Solyc04g009530 | Glutathione S-transferase | 1.60586 |
| Solyc09g011590 | Glutathione S-transferase-like protein | 3.15723 |
| Solyc06g069040 | Glutathione S-transferase | 2.6432 |
| Solyc09g011560 | Glutathione S-transferase-like protein | 1.29425 |
| Solyc07g056490 | Glutathione transferase | 1.23871 |
| Solyc07g045540 | Glucose-6-phosphate 1-dehydrogenase | 1.09805 |
| Solyc09g011650 | Glutathione S-transferase | 1.25439 |
| Solyc01g081270 | Glutathione S-transferase | 1.04629 |
| Solyc01g081250 | Glutathione-S-transferase | -3.16092 |
| Solyc10g084960 | Glutathione S-transferase-like protein | 2.36595 |
| Solyc12g011320 | Glutathione S-transferase | 1.65122 |
| **Flavonoid biosynthesis** |  |  |
| Solyc10g050160 | Caffeoyl-CoA 3-O-methyltransferase | -1.4768 |
| Solyc02g093270 | Caffeoyl-CoA O-methyltransferase | 1.89767 |
| Solyc03g115220 | Cytochrome P450 | 1.81019 |
| Solyc02g083860 | Flavanone 3-hydroxylase | 1.77739 |
| Solyc11g013110 | Anthocyanidin synthase | 1.1581 |
| **Biosynthesis of amino acids** | |  |
| Solyc10g085550 | Enolase | 1.21993 |
| Solyc11g007690 | Pyruvate kinase | 1.79015 |
| Solyc07g021630 | Branched-chain-amino-acid aminotransferase | 1.71365 |
| Solyc03g043880 | Branched-chain amino acid aminotransferase | 1.68942 |
| Solyc11g066890 | Prephenate dehydratase | -1.35096 |
| Solyc01g080280 | Glutamine synthetase | 5.92097 |
| Solyc01g110360 | Fructose-bisphosphate aldolase | 1.57975 |
| Solyc03g044330 | Acetolactate synthase | 2.38879 |
| Solyc09g008280 | S-adenosylmethionine synthase | 1.01187 |
| Solyc05g013380 | Alanine aminotransferase 2 | 1.05843 |
| Solyc07g053720 | Tyrosine aminotransferase-like protein | -1.24057 |
| Solyc12g010840 | Ketol-acid reductoisomerase | 1.38823 |
| Solyc02g062340 | Fructose-bisphosphate aldolase | 2.52241 |
| Solyc03g123830 | Phosphoglycerate dehydrogenase | 1.91969 |
| Solyc03g111000 | Glyceraldehyde 3-phosphate dehydrogenase | 1.09741 |
| Solyc09g090900 | 3-isopropylmalate dehydratase large subunit 2 | 4.31831 |
| Solyc12g089210 | Ornithine carbamoyltransferase | -1.24395 |
| Solyc08g014130 | 2-isopropylmalate synthase 1 | 1.48311 |
| Solyc06g064550 | Aspartokinase-homoserine dehydrogenase | 1.41906 |
| Solyc06g050630 | Prephenate dehydrogenase family protein | 2.77821 |
| **Arginine and proline metabolism** | |  |
| Solyc02g086970 | Aldehyde dehydrogenase 1 | -1.96627 |
| Solyc04g026030 | Spermidine synthase | -1.24424 |
| Solyc04g081930 | Prolyl 4-hydroxylase alpha-2 subunit | -1.14644 |
| Solyc02g089630 | Proline dehydrogenase | 2.12354 |
| Solyc01g080280 | Glutamine synthetase | 5.92097 |
| Solyc05g052100 | Glutamate dehydrogenase | -1.55238 |
| Solyc10g054440 | Arginine decarboxylase | 2.98153 |
| Solyc03g114150 | Aldehyde dehydrogenase | 1.98487 |
| Solyc05g005700 | Aldehyde dehydrogenase 1 | 1.92107 |
| Solyc12g089210 | Ornithine carbamoyltransferase | -1.24395 |
| Solyc06g060250 | Aldehyde dehydrogenase family protein expressed | 1.0453 |
| **Phenylalanine metabolism** | |  |
| Solyc04g064690 | Peroxidase | 3.18642 |
| Solyc08g079430 | Primary amine oxidase | -1.34109 |
| Solyc02g092580 | Peroxidase | 4.52058 |
| Solyc04g071890 | Peroxidase 4 | 3.30134 |
| Solyc02g094180 | Peroxidase 1 | 3.69003 |
| Solyc10g050160 | Caffeoyl-CoA 3-O-methyltransferase | -1.4768 |
| Solyc07g053720 | Tyrosine aminotransferase-like protein | -1.24057 |
| Solyc02g079500 | Peroxidase | 2.44229 |
| Solyc05g052280 | Peroxidase | 3.73716 |
| Solyc09g007520 | Peroxidase | 1.70618 |
| Solyc10g086180 | Phenylalanine ammonia-lyase | 1.20636 |
| Solyc03g025380 | Peroxidase | 3.70633 |
| Solyc02g093270 | Caffeoyl-CoA O-methyltransferase | 1.89767 |
| Solyc01g105070 | Peroxidase | -2.58859 |
